# SBOannotator: a Python tool for the automated assignment of systems biology ontology terms

**DOI:** 10.1093/bioinformatics/btad437

**Published:** 2023-07-14

**Authors:** Nantia Leonidou, Elisabeth Fritze, Alina Renz, Andreas Dräger

**Affiliations:** Computational Systems Biology of Infections and Antimicrobial-Resistant Pathogens, Institute for Bioinformatics and Medical Informatics (IBMI), Eberhard Karl University of Tübingen, 72076 Tübingen, Germany; Department of Computer Science, Eberhard Karl University of Tübingen, 72076 Tübingen, Germany; German Center for Infection Research (DZIF), partner site Tübingen, Germany; Cluster of Excellence ‘Controlling Microbes to Fight Infections,’ Eberhard Karl University of Tübingen, 72076 Tübingen, Germany; Department of Computer Science, Eberhard Karl University of Tübingen, 72076 Tübingen, Germany; Computational Systems Biology of Infections and Antimicrobial-Resistant Pathogens, Institute for Bioinformatics and Medical Informatics (IBMI), Eberhard Karl University of Tübingen, 72076 Tübingen, Germany; Department of Computer Science, Eberhard Karl University of Tübingen, 72076 Tübingen, Germany; Cluster of Excellence ‘Controlling Microbes to Fight Infections,’ Eberhard Karl University of Tübingen, 72076 Tübingen, Germany; Computational Systems Biology of Infections and Antimicrobial-Resistant Pathogens, Institute for Bioinformatics and Medical Informatics (IBMI), Eberhard Karl University of Tübingen, 72076 Tübingen, Germany; Department of Computer Science, Eberhard Karl University of Tübingen, 72076 Tübingen, Germany; German Center for Infection Research (DZIF), partner site Tübingen, Germany; Cluster of Excellence ‘Controlling Microbes to Fight Infections,’ Eberhard Karl University of Tübingen, 72076 Tübingen, Germany

## Abstract

**Motivation:**

The number and size of computational models in biology have drastically increased over the past years and continue to grow. Modeled networks are becoming more complex, and reconstructing them from the beginning in an exchangeable and reproducible manner is challenging. Using precisely defined ontologies enables the encoding of field-specific knowledge and the association of disparate data types. In computational modeling, the medium for representing domain knowledge is the set of orthogonal structured controlled vocabularies named Systems Biology Ontology (SBO). The SBO terms enable modelers to explicitly define and describe model entities, including their roles and characteristics.

**Results:**

Here, we present the first standalone tool that automatically assigns SBO terms to multiple entities of a given SBML model, named the SBOannotator. The main focus lies on the reactions, as the correct assignment of precise SBO annotations requires their extensive classification. Our implementation does not consider only top-level terms but examines the functionality of the underlying enzymes to allocate precise and highly specific ontology terms to biochemical reactions. Transport reactions are examined separately and are classified based on the mechanism of molecule transport. Pseudo-reactions that serve modeling purposes are given reasonable terms to distinguish between biomass production and the import or export of metabolites. Finally, other model entities, such as metabolites and genes, are annotated with appropriate terms. Including SBO annotations in the models will enhance the reproducibility, usability, and analysis of biochemical networks.

**Availability and implementation:**

SBOannotator is freely available from https://github.com/draeger-lab/SBOannotator/.

## 1 Introduction

Ontologies are used to share common knowledge and its application across communities (Stevens *et al.* 2000). While concepts in biology are adequately covered by appropriate ontologies, model-related semantics are encoded by standardized Systems Biology Ontology (SBO) terms (Courtot *et al.* 2011). The SBO is a set of orthogonal controlled vocabulary terms used to explicitly and unambiguously describe the semantics of model instances. They are divided into eight orthogonal vocabularies and can be employed to annotate a model and describe various entities. For instance, they may represent the type or role of a single component in a model streamlining the understanding and meaning of this entity. The more specific the SBO term is, the more precise the description. As of January 2023, they consist of 694 terms, with 24 newly added in the last 3 years. Generally, such terms ensure model reproducibility and exchangeability as they record and categorize the semantics of model components. From the release of SBML Level 2 Version 2 in the fall of 2006 to the current edition (SBML Level 3 Version 2 Release 2, Hucka *et al.* 2019), the SBML format has supported annotating its components using SBO terms to unambiguously mark their semantics and extend their scope. At this point, adding general, top-level SBO terms to a model can be done automatically. However, adding precise descriptions for biochemical reactions in constraint-based models, e.g. glycosylation or hydrolysis, remains a laborious and complicated step. After precise categorization, all terms must be determined and added individually to each occurrence. A higher-level SBO term specificity in reactions can enable new model analysis methods. For instance, similar to the gene set enrichment analysis, by counting the occurrence of SBO terms, one could easily deduce the types of over-catalyzed reactions, either for complete models or selected pathways. Hence, their automated assignment is of great importance. Here, we implemented an expert knowledge-driven classification scheme implemented in Python called SBOannotator, which can be easily used to assign SBO terms in a given SBML model (Keating *et al.* 2020) automatically.

## 2 Results

**Figure 1.**
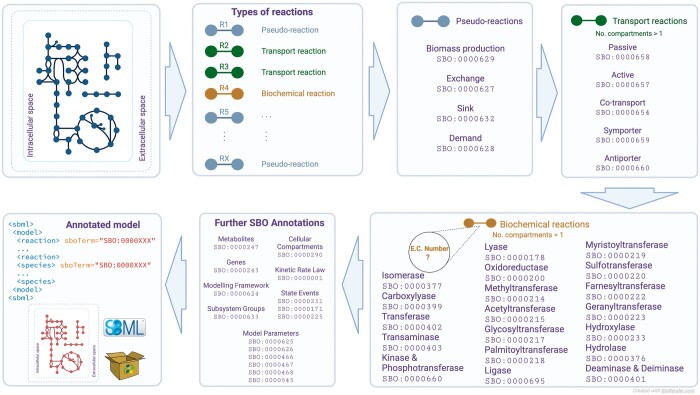
Overview of SBOannotator’s pipeline. The software enables the automated assignment of SBO terms to multiple model entities in a given SBML file. First, the pseudo- and transport reactions are characterized and classified. Then, the biochemical reactions are subdivided into 18 classes based on the underlying enzymatic function. Finally, further model elements are annotated by their respective SBO annotations, and the final model is stored in SBML format. The main advantage is the detailed categorization of biochemical reactions and the allocation of specialized terms that precisely capture related and exchangeable information.

The SBOannotator workflow comprises six main steps (Fig. 1). At first, all reactions found within the model are labeled as either (i) *transporters* that move molecules across different compartments, (ii) *simple biochemical reactions* that only take place in the cytosol, and (iii) *pseudo-reactions* that import or export metabolites and serve modeling purposes. Pseudo-reactions in systems biology modeling do not correspond to any actual physical process and should not be confused with the pseudo-first-order reactions from the field of chemical kinetics. They are subdivided into demand, exchange, and sink reactions. The biomass objective function also belongs to this class. SBOannotator processes further by examining the transport reactions and assigning appropriate SBO terms. The classification mechanism in this step is comparably advanced since several types of transporters exist. The decision relies on the main characteristics of the different classes, such as the presence of one (passive transport) or more reaction participants, and the consumption of adenosine triphosphate (ATP) or phosphoenolpyruvate (PEP) (active transport). The remaining biochemical reactions are processed in the next step to enable more detailed labeling. For this purpose, the SBOannotator employs an Structured Query Language (SQL) database that contains mappings between Enzyme Commission (EC) numbers and the respective SBO terms. As the model’s size increases, using an already-defined database accelerates the computational time needed for their annotation. Our mappings could be divided into three main categories: (i) one-to-one mapping; one SBO term represents EC numbers from a single sub-subclass (e.g. transamination); (ii) one-to-few mapping; one SBO term maps only a subset of EC numbers belonging in a single sub-subclass (e.g. myristoylation); and (iii) one-to-many mapping; one SBO term covers a large subset of EC numbers within one sub-subclass (e.g. acetylation). Supplementary Table S1 lists all mappings in detail. It is important to note that a proper term that describes the ligases (EC class 6) was missing from the SBO graph. This would be necessary to describe, for instance, reactions involving the formation of deoxyribonucleic acid (DNA), ribonucleic acid (RNA), and protein fragments. After contacting the developers of the SBO vocabulary, a new term was introduced (16 May 2023) for ligases that describes the formation of a covalent bonds (SBO:0000695). The SBOannotator is designed to handle models with or without EC numbers assigned. However, they should utilize the Biochemical, Genetical, and Genomical (BiGG, Norsigian *et al.* 2020) identifiers. If the input model provides no EC numbers, an integrated Application Programming transfer Interface (API) call requests the necessary information from the BiGG database and adds all missing annotations into the model. Depending on the model’s size, this step may increase the computational time. Hence, we recommend the prior use of an annotation tool, such as ModelPolisher (Römer *et al.* 2016). We have tested the performance of SBOannotator in assigning descriptive and more precise terms to biochemical reactions using 108 metabolic models from the BiGG database. All downloaded models contained only five types of SBO annotation representing only top-level terms. The biochemical reactions made up the largest group before and after the SBOannotator (see Supplementary Tables S2 and S3). However, their coverage was reduced from 57.9% to 18.9%, meaning a large percentage of the initial reactions got a more specific term (see Supplementary Figs S1 and S2). Finally, SBOannotator assigns SBO terms to the remaining model entities, such as metabolites and genes. The final annotated SBML model is stored in the current directory with the tag _SBOannotated.

## 3 Conclusion

Unlike other annotations, like the EC numbers, the SBO terms are directly assigned in SBML (Keating *et al.* 2020) as individual attributes to any model element. This results in a direct one-to-one mapping that indicates the function of that element inside the model. In contrast, numerous EC numbers and further references may be provided to the same element to show the link between an annotation and a model element, each with different qualifiers. For instance, a reaction in SBML may involve a so-called modifier in addition to reactants and products. This could be an inhibitor, an (enzymatic) catalyst, or some other stimulator. If multiple EC numbers link a modifier to a specific enzyme, the SBOannotator can interpret this information and add a precise SBO term for “enzymatic catalyst”. Without specifying the exact mechanism of this catalysis, the role of the modifier is now defined through an “is a”-relationship: this modifier is an enzymatic catalyst. Once assigned, other software, like the SBMLsqueezer 2 (Dräger *et al.* 2015), may interpret this information to automatically derive suitable reaction rate laws, where it is of great importance if the modifier is an inhibitor or an enzyme. With this, the information from the EC numbers becomes directly accessible and interpretable: instead of a potentially extensive list of various annotations, there is a single attribute with a defined value. By this, SBOannotator helps to define more clearly which role individual elements play within a model.

Overall, the SBOannotator is a freely available and user-friendly Python tool. It can be easily employed to rapidly annotate systems biology metabolic networks in SBML format with appropriate SBO terms, with particular emphasis on allocating precise and descriptive terms to all chemical reactions. The minimal requirement for the tool is a valid SBML format of the input model(s). SBOannotator will then proceed with the labeling of model components with terms based on the defined model entities and attributes. The assignment of enzyme-based SBO terms to reactions is hinged upon the existence of standardized BiGG (Norsigian *et al.* 2020) identifiers. However, expanding the usability of SBOannotator by enabling the utilization of further database identifiers to extract enzymatic information would be of great importance. So far, SBOannotator is a standalone application. Its integration into existing software, such as ModelPolisher (Römer *et al.* 2016), could be worthwhile, as long as an abstract use of the tool by the users is possible. Lastly, since the main emphasis of the tool is the precise annotation of biochemical reactions, it has been developed on the basis of genome-scale metabolic models. However, SBO terms may also be useful in other modeling frameworks, such as, the automated assignment of rate laws for dynamic simulations. Hence, the SBOannotator could be extended to assign additional terms specific to different model types, including dynamic, stochastic, and population models.

## Supplementary Material

btad437_Supplementary_Data

## Data Availability

The SBOannotator tool, all related data, and a demo script to run the code are available in a git repository at https://github.com/draeger-lab/SBOannotator/. Along with this article, a supplementary table in Comma-separated Values (CSV) format is available.
