# Supplementary figures and images for: SBOannotator: a Python tool for the automated assignment of systems biology ontology terms
Source: Bioinformatics. 2023 Jul 14;39(7):btad437. doi: 10.1093/bioinformatics/btad437 (PMC10371491; doi:10.1093/bioinformatics/btad437)

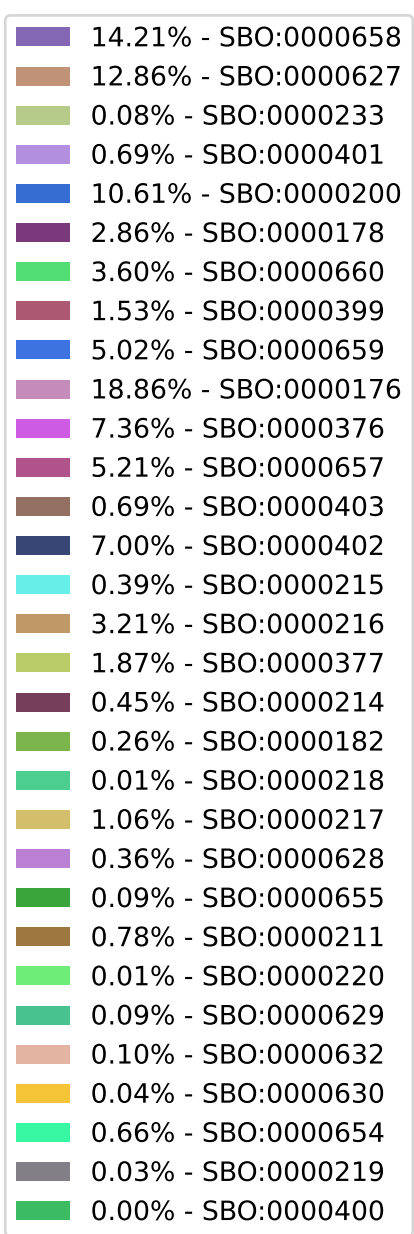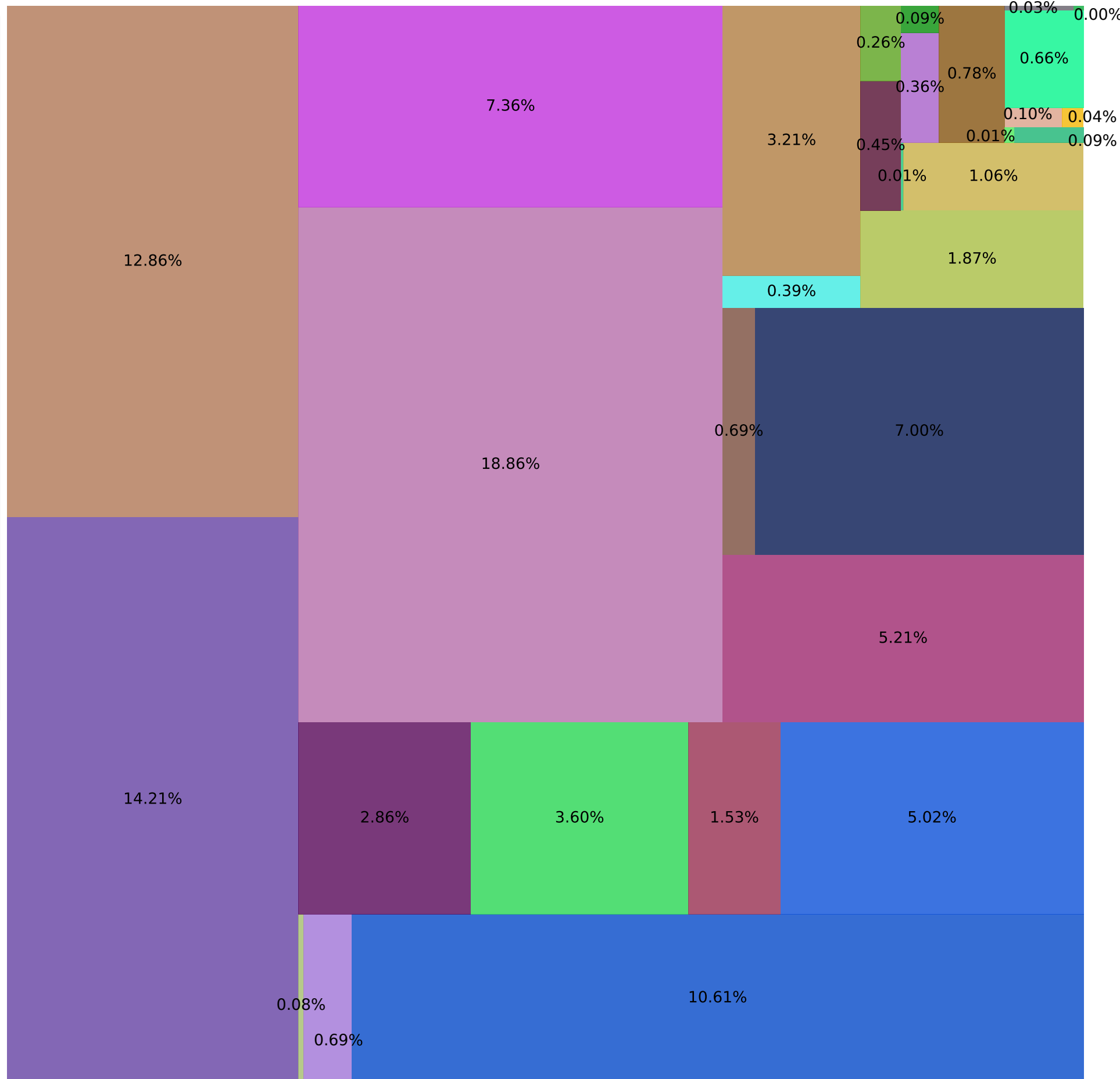

Supplement: btad437_Supplementary_Data [file btad437_supplementary_data.zip › S2_Figure.pdf]

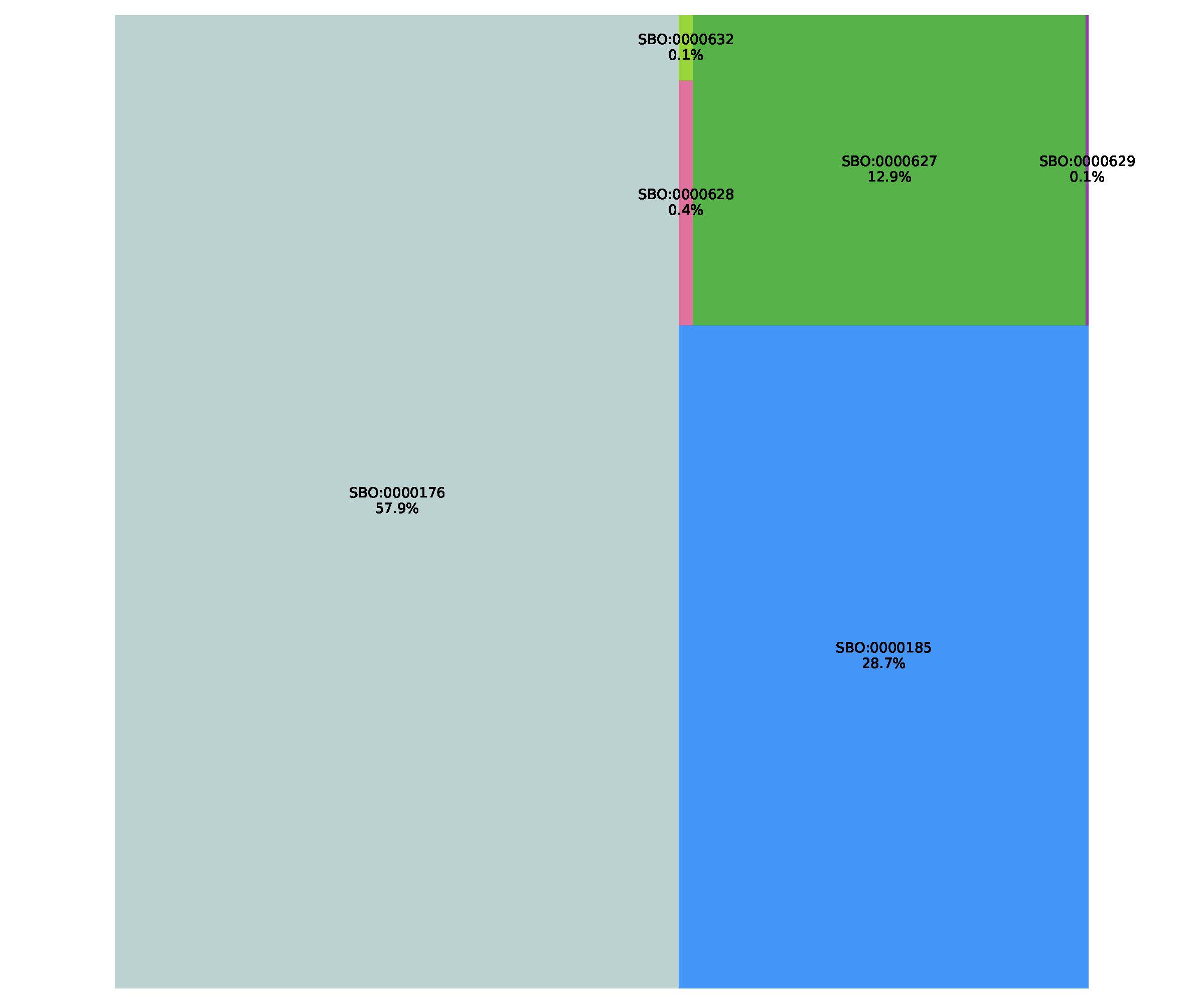

Supplement: btad437_Supplementary_Data [file btad437_supplementary_data.zip › S1_Figure.jpg]
