# Supplementary material for: SBOannotator: a Python tool for the automated assignment of systems biology ontology terms
Source: Bioinformatics. 2023 Jul 14;39(7):btad437. doi: 10.1093/bioinformatics/btad437 (PMC10371491; doi:10.1093/bioinformatics/btad437)
Supplement: btad437_Supplementary_Data [file btad437_supplementary_data.zip › ATT00001.htm]

> On 12. Jul 2023, at 06:23, Bioinformatics <onbehalfof@manuscriptcentral.com> wrote:
>
>   
>
> 12-Jul-2023  
>   
> BIOINF-2023-0306.R2 - SBOannotator: a Python Tool for the Automated Assignment of Systems Biology Ontology Terms  
>   
> Dear Prof. Dräger,  
>   
> Thank you for providing the updated files.  
>   
> This is to confirm that I successfully uploaded the updated files in the system. But before I go ahead and proceed your paper to the production, may I request for an updated copy of one of your supplementary files with no overlapping characters? You my attach to this email the updated file and I will be glad to upload it in your behalf.  
>   
> Please see attached file for your reference.  
>   
> Sincerely,  
>   
> Ms. Margarette Mhay Esquivias  
> Bioinformatics  
> bioinformatics.veo@oup.com<S2-Figure.jpg>

  
  

With best regards

Dr. Andreas Draeger

Assistant Professor

---

Eberhard Karls University Tübingen

Institute for Bioinformatics and Medical Informatics (IBMI)

Computational Systems Biology of Infections and Antimicrobial-Resistant Pathogens  
Sand 14 · Office #C108 · 72076 Tübingen · Germany  
Phone: +49-7071-29-70459 · Fax: +49-7071-29-5152

Web: http://systems-biology.info · Twitter: @dr\_drae

YouTube: https://www.youtube.com/c/systemsbiology
